# Opioid misuse detection from cognitive and physiological data with temporal fusion deep learning

**DOI:** 10.1016/j.drugalcdep.2025.112774

**Published:** 2025-07-08

**Authors:** Bhanu Gullapalli, Yunfei Luo, Tauhidur Rahman, Eric L. Garland

**Affiliations:** a Halıcıoğlu Data Science Institute, Department of Computer Science and Engineering, University of California San Diego, United States; b Department of Psychiatry, University of California San Diego, La Jolla, CA, United States; c Sanford Institute for Empathy and Compassion, University of California San Diego, La Jolla, CA, United States

**Keywords:** Addiction, Chronic pain, Machine learning, Pain, Psychophysiology

## Abstract

**Introduction:**

Machine learning may enable detection of opioid misuse to prevent opioid-related risks including overdose and opioid use disorder.

**Methods:**

Here, we collected 9238 datapoints from on-body sensors and cognitive tasks in a sample of 169 patients who were prescribed opioid analgesics to manage chronic pain. We categorized patients into one of two groups using the Current Opioid Misuse Measure (COMM): those showing signs of opioid misuse (MISUSE+, n = 116) and those without signs of opioid misuse (MISUSE−, n = 53). Heart rate variability and respiration rate were assessed while participants completed a Dot Probe task involving shifting attention towards and away from opioid-related and emotional cues, and a Go/No-Go task involving inhibition of automatic responses. Cross-sectional data (e.g., physiological responses, task reaction times, task accuracy) were analyzed with a temporal fusion transformer machine learning (ML) model to predict COMM opioid misuse status. We employed Leave-One-Group-Out (LOGO) cross-validation with the participants divided into 10 groups. Each cycle, one group was held out for testing, ensuring robust, unbiased model validation across different subsets of participants.

**Results:**

The ML model showed good predictive performance for identifying opioid misuse (AUC, 0.81; specificity, 0.78; sensitivity, 0.78). Behavioral responses were stronger predictors of misuse status than physiological signals.

**Conclusions:**

ML models using data from cognitive tasks and on-body sensors detected opioid misuse with an accuracy comparable to gold-standard self-reported opioid misuse assessments. Wearable sensors may provide only incremental predictive power over behavioral responses. Our ML model should be benchmarked against objective measures of opioid misuse.

## 1. Introduction

Long-term opioid analgesic use increases risk for opioid misuse which has impacted public health worldwide, with deleterious effects including risk for suicide and other mental health conditions, overdose, and development of opioid use disorder (OUD) (Chou et al., 2015; Madras, 2017). Digital healthcare and machine learning integration offer new avenues for diagnosis and predictive models for opioid risk management (Carreiro et al., 2020; Gullapalli et al., 2021; Miller-Rosales et al., 2023; Murray et al., 2016). Currently, self-report methods (e.g., questionnaires and clinical interviews) are among the most common means of identifying opioid misuse (Lawrence et al., 2017). However, given that disclosure of opioid misuse may result in forced opioid tapering or termination of opioid prescribing, self-reports of substance misuse are subject to social desirability bias and deception (Fishbain et al., 1999; Sanders et al., 2016). In contrast, objective markers obtained through cognitive tasks and on-body sensors may overcome these limitations.

To date, cognitive tasks that assess attentional bias and deficits in response inhibition have shown some degree of promise in the detection of addictive behavior (Garland and Howard, 2014; Yang et al., 2009). Attentional bias is the tendency to preferentially attend to an emotionally salient stimulus (e.g., an image of an opioid pill) over a neutral stimulus, as evidenced by comparatively speeded reaction times (RTs) and physiological reactivity on computerized tasks like the Dot Probe task, on which participants shift attention towards and away from emotionally-salient cues to identify the location of a target a probe. In contrast, response inhibition is reflected by the extent to which one can inhibit a habitual behavioral response, as evidenced by errors of commission on the Go/No-Go task when a signal to stop the response is given after an automatic response set has been established through frequent button pressing (Chambers et al., 2009). These two cognitive mechanisms are theorized to underlie craving and impaired self-control – key drivers of addictive behavior (Verdejo-Garcia et al., 2019). Yet, the reliability and accuracy of these measures has been questioned (Rodebaugh et al., 2016; Zorowitz and Niv, 2023).

The dynamic nature of physiological signals and cognitive responses necessitates advanced modeling techniques to unravel the intricacies of opioid misuse behaviors. Traditional frequentist statistics may not comprehensively leverage the “big data” produced by cognitive tasks and psychophysiology. In contrast, our study adopts a temporal fusion-based multivariate machine learning approach, analyzing data across multiple timescales to improve the detection of opioid misuse. Machine learning of electronic healthcare data has already begun to demonstrate utility in detection of opioid misuse and OUD (Garbin et al., 2023; Liu et al., 2023); however the application of machine learning models to identify cognitive and physiological markers of opioid misuse remains underexplored.

We utilize a carefully curated dataset of 169 chronic pain patients, encompassing both individuals displaying aberrant behaviors indicative of opioid misuse as well as individuals who took opioids as prescribed by their physicians. Our approach involves a Temporal Fusion Transformer (TFT) (Lim et al., 2021) model to categorize opioid misuse. This model leverages attention mechanisms (Li et al., 2019; Vaswani et al., 2017) that dynamically assess the relevance of inputs for predictions, assigning temporal significance to physiological and behavioral signals. Here we advance a reproducible and validated artificial intelligence (AI)-based approach to predictive modeling of opioid misuse risk, with implications for detecting treatment response.

## 2. Methods

### 2.1. Participants

Eligible participants were ≥ 18 years, English-speaking, and had been using prescription opioids daily for > 3 months (identified by electronic medical record review and self-report). Participants were recruited from primary and specialty care clinics, electronic medical record review, physician referrals, community outreach, and advertisements between April 2012 and March 2020. The institutional review boards of Florida State University and University of Utah approved the protocol.

### 2.2. Procedures

After informed consent, participants were assessed with demographic and clinical measures, including the Current Opioid Misuse Measure (COMM), a well-validated and broadly used measure of aberrant drug-related behaviors associated with opioid misuse (Butler et al., 2007). The original COMM validation study conducted with patients treated in specialty pain management clinics found that a score of ≥ 9 was suggestive of prescription opioid misuse (Butler et al., 2007). However, according to a study of a broad sample of chronic pain patients from a variety of primary care settings who were prescribed long-term opioid therapy, receiver–operator characteristic curve analyses revealed that a score of 13 or higher on the COMM had maximum sensitivity (0.77) and specificity (0.77) to identify opioid misuse behaviors consistent with disordered opioid use among chronic pain patients in primary care settings (Meltzer et al., 2011). We chose this more conservative COMM cut-off value (≥13) as our criterion value of opioid misuse to minimize false positives and because, similar to Meltzer et al. (2011), our sample was not confined to patients drawn from specialty pain clinics. Participants with COMM scores ≥ 13 were classified as showing signs of opioid misuse (MISUSE+), whereas those with COMM scores < 13 were classified as not showing signs of misuse (MISUSE−). Binary classification of opioid misuse among patients on long-term opioid therapy has been shown to reliably distinguish MISUSE+ from MISUSE− groups on a range of psychophysiological variables (Garland et al., 2017; Garland and Howard, 2021; Hudak et al., 2022, 2021). Such classification has real world clinical relevance, in that clinicians need to know whether a given patient has exceeded a validated threshold for risk of opioid misuse to inform their clinical decision making. Our recruitment strategies resulted in an approximate 2:1 ratio of MISUSE+ vs. MISUSE− participants. Participants were briefed on cognitive task procedures and equipped with on-body sensors (BIOPAC MP150) that captured electrocardiogram (ECG), and respiration data, all sampled at a rate of 2000 Hz. Upon completing the study, participants were compensated.

#### 2.2.1. Cognitive task: dot probe

A Dot Probe task measured attentional bias (Field and Cox, 2008). Each trial began with a fixation cross for 500 ms. Next, two images matched for visual complexity, composition, and figure-ground relationships appeared side-by-side on a computer screen for either 50, 200, or 2000 ms. Three blocks of cues (opioid-related, pain-related, and pleasure-related) were presented in randomized, counterbalanced order. Three sets of 12 photographs, each set representing one type of cue, were selected from the International Affective Picture System (IAPS) (Bradley and Lang, 2017) and online media libraries. Opioid-related cues included images of pills and pill bottles. Pain-related cues included images of injuries, painful medical procedures, and faces grimacing in pain. Pleasure-related cues included images of romantic couples, athletic victories, and food. A set of 36 neutral images was selected from the IAPS and each neutral image was paired with an emotionally-salient image. Presentation duration and left/right position of the images were randomized and counterbalanced within each block of 96 trials. Image pairs disappeared, and a target probe randomly replaced one of the images for 100 ms. Participants indicated the location of the target with a left/right button press, and RT was recorded.

#### 2.2.2. Cognitive task: emotional Go/No-Go

An Emotional Go/No-Go task (Albert et al., 2010) measured response inhibition. Go and No-Go stimuli consisted of two capital letters (“M” and “W” respectively) superimposed over 12 images used as background contexts (3 neutral, 3 opioid-related, 3 pain-related, and 3 pleasure-related images). Participants were instructed to press a button whenever the letter “M” (Go) was presented, and to withhold pressing the button when the letter “W” (NoGo) was presented. The order in which the image contexts were presented was randomized and counterbalanced. Each context contained 133 letters (93 Go and 40 NoGo) presented in three blocks. Go and NoGo trials were presented in semi-random order within each block, such that NoGo trials could be preceded by one-to-four Go trials and there was never a consecutive presentation of two NoGo trials. Trials began with the presentation of the letter M or W (200 ms), followed by a fixation cross (800 ms); 500 ms later, the next letter appeared.

**Fig. 1.**
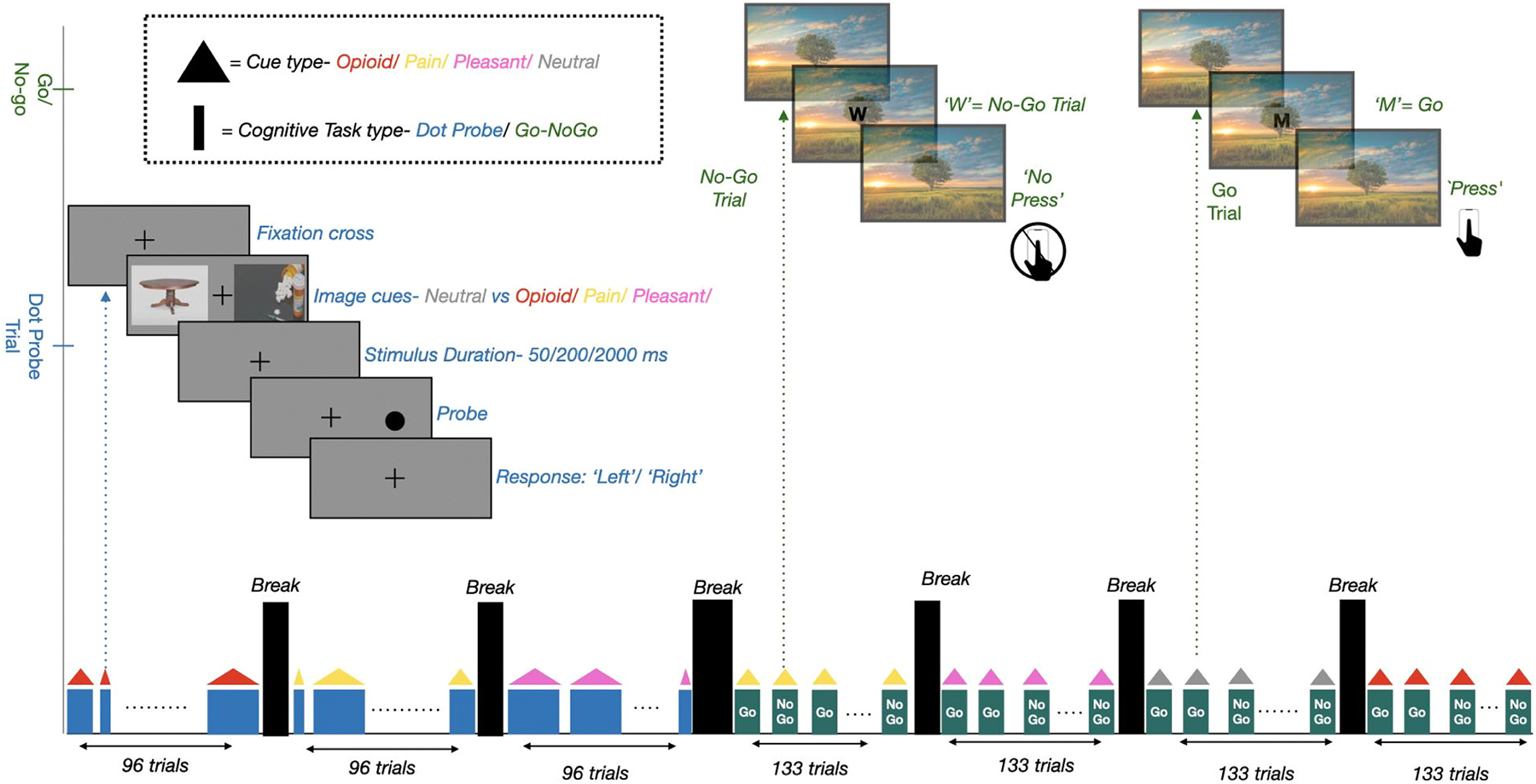
Depictions of the Dot Probe and Go/No-Go tasks used in this study.

The Dot Probe and Go/No-Go tasks were programmed using E-Prime 2.0 software(E-Prime^®^, Psychology Software Tools, 2017) (see Fig. 1).

### 2.3. Data preprocessing and handling missing values

Missing data arose from two main sources: 1) signal artifacts in physiological measurements and 2) loss of behavioral data from non-responses in the task. Signal artifacts (32 out of the 9238 data points: 4091 from the Dot Probe, and 5147 from the Go/NoGo task) were identified and removed prior to machine learning using Python’s Neurokit library. For ECG signals, we implemented a preprocessing pipeline to clean the data. First, we applied a filter (0.5 highpass Butterworth, order = 5) to remove slow variations in the baseline, then we removed electrical noise from power sources to increase signal clarity. Respiration signals were preprocessed using a second-order 0.05–3 Hz bandpass Butterworth filter to isolate the frequency range of normal breathing patterns while removing noise and artifacts. These filtering parameters were chosen based on established guidelines in psychophysiological research (Cacioppo et al., 2007). The number of artifacts removed represented only 0.34 % of the total dataset, suggesting high overall data quality. We made a deliberate methodological choice to retain trials with behavioral non-responses in the machine learning model, as we hypothesized that these non-responses could be meaningful indicators of attentional bias or cognitive processing patterns potentially associated with opioid misuse risk.

### 2.4. Statistical analysis

Analyses were conducted to detect differences in physiological data and cognitive features due to cue-type and participant label (MISUSE+ vs MISUSE−). We performed a 2-way ANOVA to examine the effects of cue type and opioid use class (independent variables) on features derived from physiological and cognitive data. For the continuously measured wearable data, we computed single summary statistics (e.g., mean heart rate) from each measurement window to enable traditional statistical analysis. While these analyses are reported in eTable1A and 1B of Supplement, it is important to note that this traditional frequentist approach may fail to capture the complex, nonlinear temporal patterns inherent in the rich psychophysiological and cognitive task data. This limitation of conventional statistical methods underscores the need for more sophisticated analytical approaches capable of leveraging the temporal dynamics present in our dataset.

### 2.5. Model development

**Fig. 2.**
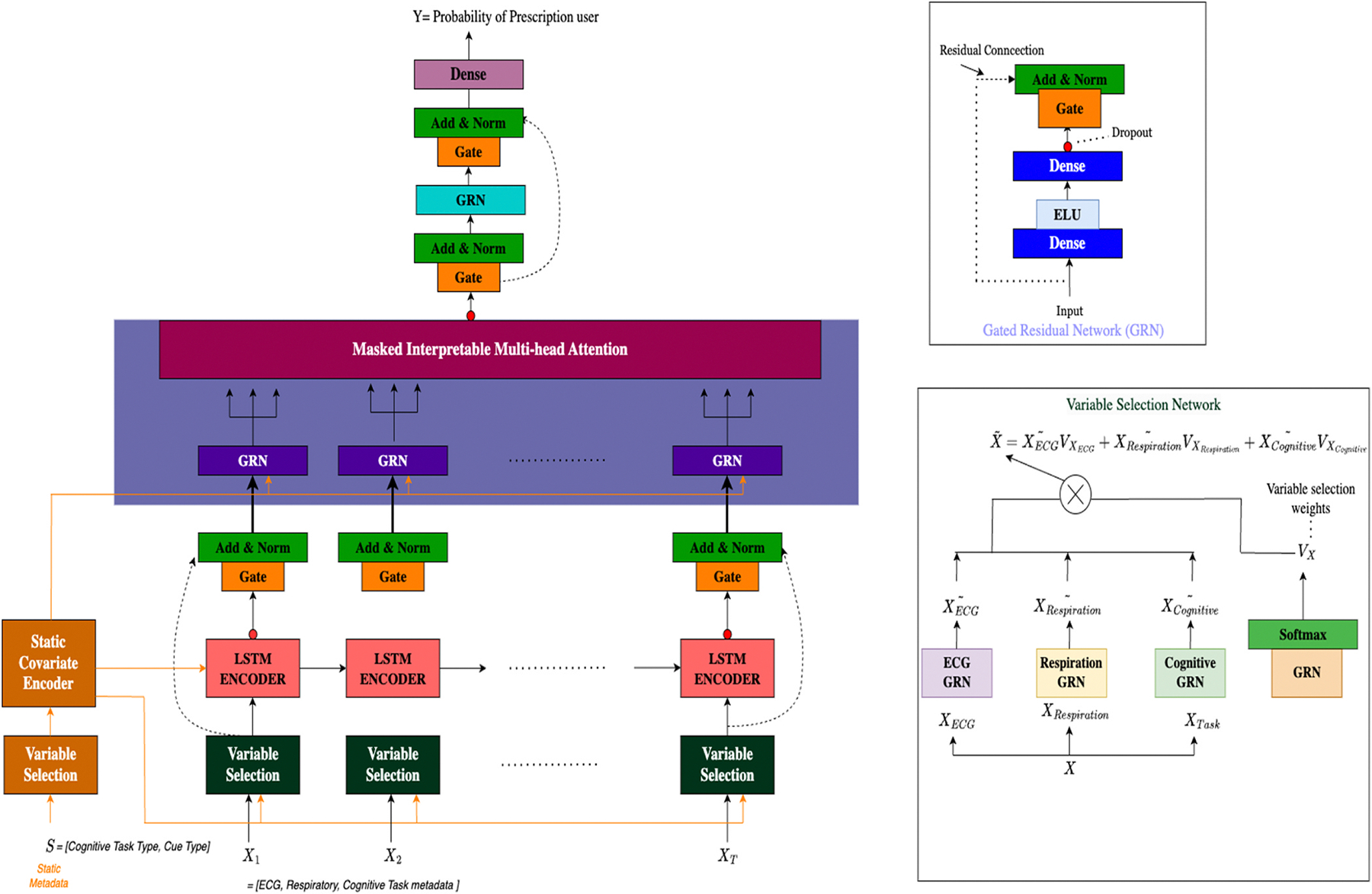
Temporal Fusion Transformer (TFT) model architecture for classifying user’s opioid usage class using cognitive and on-body sensory data.

Physiological data is continuous, raw, and exhibits multi-modality, derived from a range of on-body sensors. In contrast, behavioral data from cognitive tasks is event-based, static (e.g., type of image cue, duration of cue presentation, task type), and possesses a much lower dimensionality compared to the data from wearables. To merge these distinct data streams of static and time varying covariates, we employed the Temporal Fusion Transformer (TFT) model (Lim et al., 2021) to predict an individual’s probability of being MISUSE− (and consequently, MISUSE+). Fig. 2 shows the model architecture in detail.

### 2.6. Model training strategies

In line with best practices in in time-series analysis (Lim et al., 2021), we trained the TFT model utilizing a 45-second time windowed wearable and cognitive data, with a sliding window of 20 s, to classify between MISUSE+ and MISUSE− groups. Studies utilizing TFT and other deep learning models for time-series classification employ sliding windows to improve predictive accuracy for predicting outcomes at single and multiple timepoints (Bai et al., 2025; Chen et al., 2022; Kim and Hong, 2024; Jiang et al., 2024; Yao et al., 2023; Weidman et al., 2025). Using overlapping windows increases the effective sample size, allowing the model to learn more robust temporal patterns rather than relying on a single grand average (collapsed across all trials) per participant. Further, behavioral and physiological responses evoked by the Dot probe and Go/NoGo tasks fluctuate dynamically over the duration of the tasks as a function of the different stimuli, stimulus durations, and instructions (e.g., to deploy or withhold a button press) for each randomly presented trial type. A single prediction based on an entire dataset may obscure meaningful short-term variations in psychophysiological responses, whereas multiple overlapping windows allow the model to detect dynamic shifts in behavior and physiology predictive of opioid misuse. We tested window sizes of [15, 45, 90, 180] seconds, treating it as a hyperparameter. The window size of 45 s yielded the best classification accuracy. Each 45 s window covers approximately 10–20 trials of Go/NoGo and Dot probe tasks. The window size of 45 s comprising approximately 10–20 trials, which is a reasonable amount of information for the model to learn the relationship between the subject’s behavior and the misuse risk.

Our final model outputs the prediction probability of MISUSE− classification (as the dataset contained more MISUSE+ than MISUSE−) for each of these windows and is trained using weighted cross entropy loss to adjust for the class imbalance. We employed a leave-one-group-out (LOGO) cross-validation method with 10 folds, where all participants were first randomly divided into 10 mutually exclusive groups. For each iteration of cross-validation, one group was held out as the test set while the remaining nine groups were used for training, following the LOGO validation principle. The groups were stratified to maintain the same ratio of MISUSE+ to MISUSE− across all folds. The prediction probabilities from these 45-second time windows are averaged to assign a single probability for MISUSE− and MISUSE+ classification for each participant, and we use this approach for all the analysis. The model’s performance was evaluated based on specificity, sensitivity, and AUC-ROC score.

We implemented the TFT model using Python version 3.9, Scikit-learn version 1.0.2 and Pytorch 2.0. Model training occurred on a Mac PC equipped with an Apple M1 Max chip that utilizes a 10-core CPU and a 16-core graphics processing unit, alongside 32 GB of random access memory. The tuning of hyperparameters that includes window-size, sliding window, kernel size, etc,. (eTable2 in Supplement) was done using a grid search randomly sampled 1000 data points, to mitigate the computational expense. We had a large set of hyperparameters to tune including window size, sliding window, kernel size, number of heads, batch size, and gradient norm, making a full grid search with LOGO cross-validation computationally infeasible. To mitigate this, we randomly sampled 1000 data points to identify faster-converging hyperparameter combinations, assuming these patterns would generalize to the full dataset. This strategy efficiently reduced hyperparameter space while keeping computational costs manageable. The hyperparameter combination that resulted in faster convergence was selected. We provide a description of the training process, and input features, and interpretability in Model Architecture in Fig. 2.

### 2.7. TFT model for opioid misuse

The input to the TFT model shown in Fig. 2 is a 45-second time window that consists of sensor and cognitive data. We represent this data as Static:-Cognitive Task type (Dotprobe/Go-NoGo) and Cue type (Opioid/Pain/.), which remain constant throughout this window; Continuous: ECG, Respiration, and Cognitive task performance (Reaction times and Error rates) that vary continuously based on the participant’s response and reaction in the study. Each second of this time window is used as a time step to the model. The input time steps are first passed through the variable selection network that extracts the most salient features from the static and time dependent covariates for the prediction. The output from this network is combined with the embedding extracted from the static data using a static covariate encoder of Gated residual network (GRN), this combined data is passed through LSTM encoder units that extracts embeddings (or feature representations) for each time step.

Embeddings from all the time steps are combined through a self-attention mechanism to learn long-term relationships across different time steps. To enable diverse and robust representations, we utilized the multi-headed attention mechanism inherent to the TFT architecture. This mechanism, a core component of the TFT model, captures temporal relationships and feature interactions. The output from the multi-headed attention is then passed through a GRN to incorporate additional temporal processing on the output embeddings. We integrated skip-connections at every aspect of the network to expedite convergence. Subsequently, the output from the GRN traverses a Fully-connected (FC) layer to compute the prediction probability of the participant belonging to the MISUSE− class. The model is trained from end-to-end using an objective loss function of weighted Cross-entropy between predicted and ground truth labels. For all the categorical variables (task type, cue type) we use entity embeddings to keep uniformity with the continuous ones.

### 2.8. Model interpretability

To enhance the interpretability of the model’s predictions, our analysis focuses on the weights within the TFT’s variable selection network. This network integrates both static and time varying covariates at each timestep, forming representations based on their relevance to the output prediction. Aligning with the methodology outlined in the TFT’s original paper, we employ the 10th, 50th, and 90th percentiles of each covariate’s sampling distribution as a measure for assessing their importance (Caldas and Soares, 2023; Phetrittikun et al., 2021). The observation of consistent patterns of importance across these multiple percentiles not only substantiates our findings but also underscores the effectiveness of this interpretive approach.

## 3. Results

### 3.1. Characteristics of participants

Our cohort study (Table 1) included 169 participants with a mean age of 50.6 years (SD, 13.3 years), consisting of 116 MISUSE+ (68.6 %) and 53 MISUSE− participants (31.4 %). Of the participants, 42 % were men and 58 % were women. Among the 169 participants, the majority were White (n = 118; 69.8 %) or African American (n = 18, 10 %). In terms of income, 106 participants (62.73 %) reported an annual income of less than $40 K. The most common chronic pain conditions reported were low back pain (n = 105, 62.1 %) and arthritis (n = 20, 11.8 %), and many patients (n = 47, 27.8 %) reported multiple chronic pain conditions. Regarding opioid use, the majority of participants were prescribed either oxycodone (n = 50, 29.5 %) or hydrocodone (n = 36, 21.3 %). The distribution of physiological and behavioral data points from each participant is shown in eFigure 1 of the supplement.

### 3.2. Experiment outcomes

**Fig. 3.**
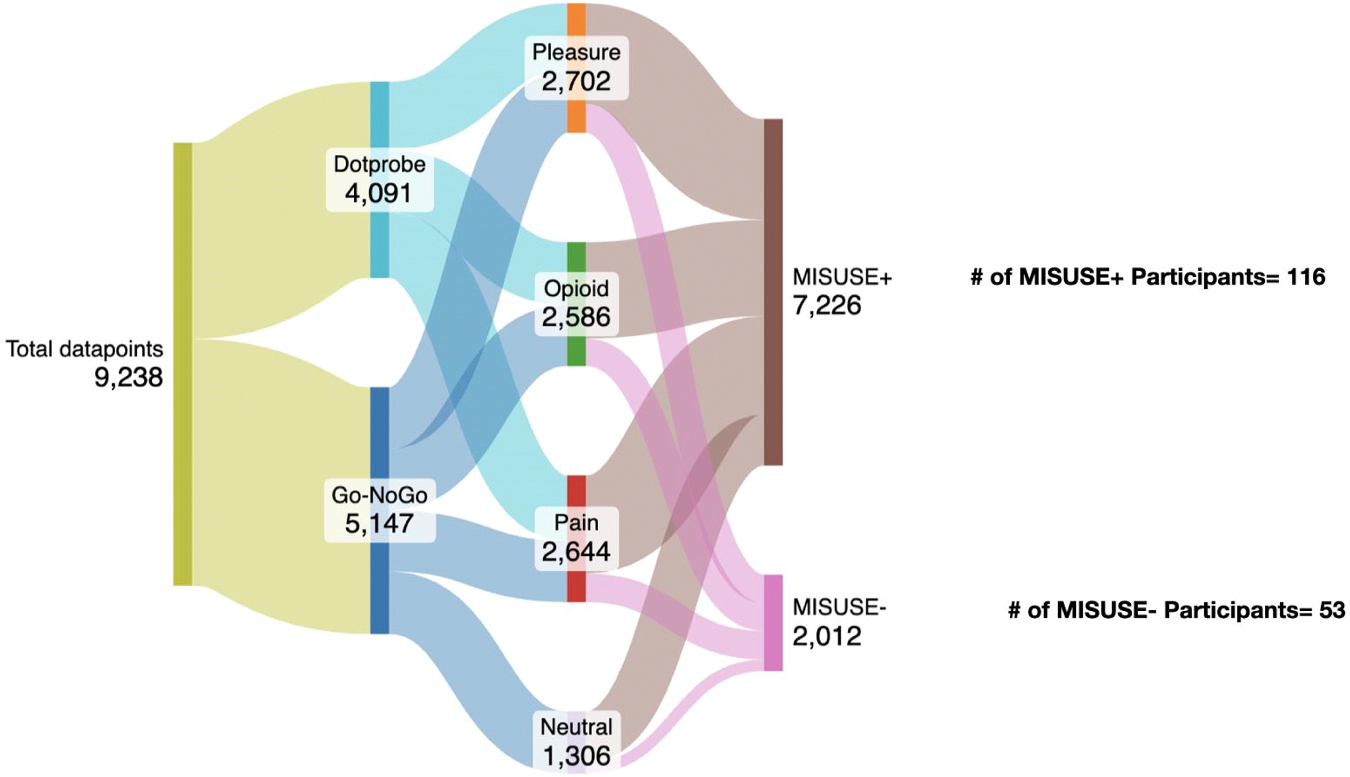
Sankey Diagram illustrating the distribution of behavioral and physiological data from cognitive tasks and the corresponding cue types used for training the Temporal Fusion Transformer: A total of 9238 data points were utilized, generated from 116 MISUSE+ participants and 53 MISUSE− user participants.

Using a 45-second time window slided by 20 s, from each participant we generated 9238 data points across the sample (see Fig. 3). TFT model, trained using a 10-fold cross-validation method, predicts the probability of MISUSE− for each data point. By calculating the average probability across each participant’s data points, we assign a label of MISUSE− (or MISUSE+), using a threshold of 0.5. Examining all data points we achieved an AUROC of 0.77 (95 % CI, 0.71–0.83), a specificity of 0.84 (95 % CI, 0.76–0.92), and a sensitivity of 0.68 (95 % CI, 0.62–0.73) with respect to misuse class. Along with TFT model we also trained and validated with other machine learning models such as Bidirectional Long Short-Term Memory (Bi-LSTM) (Graves and Schmidhuber, 2005), Gated recurrent unit (GRU) (Chung et al., 2014), and Temporal Convolution Network (TCN) (Lea et al., 2016), that are known to handle multimodal time series. The results with these models are presented in eTable 3 in supplement.

### 3.3. Predictive performance comparison with specific task and cue type

We then analyzed the differential prediction probabilities of each task and cue-type. This approach was used to determine which task and cue-type were most useful for identifying opioid misuse, and whether model performance could be enhanced. The model training remained the same as in the previous experiment, while the test data points were conditioned on cue and task type.

**Fig. 4.**
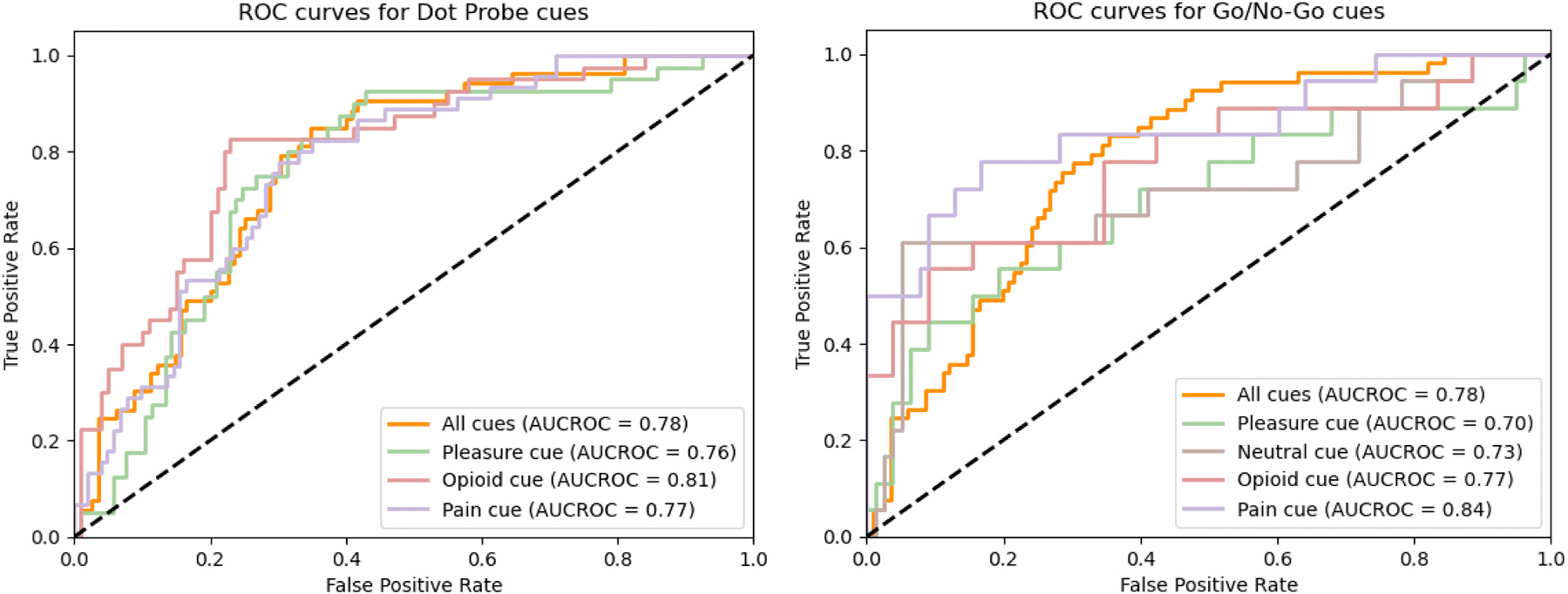
Receiver operating characteristic (ROC) curves for detecting opioid misuse classification using data points from each cue type. We combined all the results from 10-fold validation in this figure.

Focusing solely on the Dot Probe task to classify participants as MISUSE+, we examined how model performance varied across trials where opioid-, pleasure-, and pain-related cues were presented. For opioid cue trials, we observed an AUROC of 0.81 (95 % CI, 0.74–0.87), a specificity of 0.78 (95 % CI, 0.64–0.93), and a sensitivity of 0.78 (95 % CI, 0.70–0.87). For pleasure cue trials on the Dot Probe task, we achieved an AUROC of 0.74 (95 % CI, 0.67–0.82), a specificity of 0.80 (95 % CI, 0.70–0.90), and a sensitivity of 0.72 (95 % CI, 0.67–0.77). For pain cue trials, an AUROC of 0.78 (95 % CI, 0.73–0.84), a specificity of 0.78 (95 % CI, 0.67–0.89), and a sensitivity of 0.68 (95 % CI, 0.62–0.74) was observed. Overall, using opioid cues from the Dot Probe task improved the model’s performance, yielding a significantly improved sensitivity score compared to the previous experiment (p < 0.05). We reported the ROC curves from the 10-fold validation using Dot Probe cues in Fig. 4.

Similarly for the Go/No-Go task, we examined model performance for classifying MISUSE+ across trials where neutral, pleasure-related, pain-related, and opioid-related cues were presented. We observed, for neutral cue trials, an AUROC of 0.78 (95 % CI, 0.64–0.93), specificity of 0.58 (95 % CI, 0.30–0.87), and sensitivity of 0.95 (95 % CI 0.90–0.99); for pleasure cue trials, an AUROC of 0.77 (95 % CI, 0.68–0.87), specificity of 0.48 (95 % CI, 0.24–0.71), and sensitivity of 0.93 (95 % CI 0.87–0.99); for pain cue trials, an AUROC of 0.81 (95 % CI, 0.70–0.92), specificity of 0.68 (95 % CI, 0.41–0.95), and sensitivity of 0.87 (95 % CI 0.80–0.96); and for opioid cue trials an AUROC of 0.82 (95 % CI, 0.73–0.90), specificity of 0.73 (95 % CI, 0.52–0.94), and sensitivity of 0.86 (95 % CI 0.80–0.92). Opioid cue trials showed significantly greater specificity than pleasure and neutral cue trials (p < 0.05). We reported the ROC curves from the 10-fold validation using Go/No-Go cues in Fig. 4.

### 3.4. Interpreting the model using variable importance

To enhance the interpretability of the model’s predictions, our analysis focuses on the weights within the TFT’s variable selection network. The weights within the variable importance block capture the relevance for the final prediction. We interpret the model’s prediction (Table 2) by aggregating these weights and examining their values across different percentiles (10th, 50th, and 90th) (Caldas and Soares, 2023; Phetrittikun et al., 2021).

Regarding static covariates, we observed that cue-type information is significantly more important and relevant for the prediction than task-type information (p < 0.01). For continuous covariates including physiological sensor data and behavioral data, we found error rates to be most predictive of MISUSE+, followed by reaction times and ECG. Across all percentile ranges, respiration data was observed to be the least important feature. Overall, behavioral data from the cognitive tasks, especially error rates, were consistently more important than any physiological sensor data in predicting opioid misuse (p < 0.01).

## 4. Discussion

In this cohort study, our best-performing machine learning models reliably detected self-reported opioid misuse risk with an AUROC of 0.81, using on-body sensor data and objective measures from cognitive tasks that focus on attentional bias and response inhibition. Accurate detection of opioid misuse is paramount to safe opioid prescribing and prevention of serious adverse consequences of opioid use, including overdose and OUD. Cognitive tasks and on-body sensors may yield objective markers of opioid misuse risk for individuals actively seeking treatment and for those in need of interventions to prevent behavioral escalation towards full-blown OUD.

Here we used a multivariate temporal fusion machine learning model to discriminate opioid misuse from medically-appropriate opioid use among a large sample of chronic pain patients prescribed long-term opioid analgesic therapy. Findings indicate that multimodal physiological and behavioral responses collected during two simple, computerized tasks can be used to train a machine learning model to identify patients who misuse opioids with a relatively high degree of accuracy – suggesting the clinical utility of our novel approach. According to a systematic review (Lawrence et al., 2017), the most widely used screeners for opioid misuse show comparable predictive performance to our ML model: COMM (sensitivity: 0.71–0.94; specificity: 0.68–0.77); SOAPP-R (sensitivity: 0.79–0.91; specificity: 0.52–0.69). More recently, in an emergency room study, the COMM was shown to predict opioid misuse with a similar sensitivity and specificity to prescription drug monitoring program data (Wilson et al., 2020). PDMPs have been shown to have AUCs of 0.70–0.74, with similar accuracy to our machine learning system (Cochran et al., 2021). In approximately 15 min, a patient can complete the cognitive tasks with biometric sensors employed in our machine learning system. Given empirical estimates of opioid misuse prevalence among patients with chronic pain (Vowles et al., 2015), for every 1000 patients screened with the best performing system features (e.g., opioid cues on the dot probe task), 360 of these patients will be flagged at risk for misuse. Of these, around 195 would be true positives, and 165 would be false positives. Although the accuracy of our system requires further optimization, to our knowledge, this is the first demonstration of a machine learning system that detects opioid misuse risk based on multimodal psychophysiological responses from cognitive tasks.

The decision to frame opioid misuse as a binary classification task aligns with existing clinical practices and research frameworks, where individuals are commonly categorized as misusing opioids or using them as prescribed (Butler et al., 2007; Lawrence et al., 2017; Meltzer et al., 2011; Turk et al., 2008). While opioid misuse exists on a spectrum, this binary approach provides a practical and interpretable framework for clinical applications, such as screening tools and decision-support systems. Our operationalization of misuse, based on established benchmarks via COMM scores, ensures consistency with prior work and reflects clinically actionable thresholds. Nevertheless, we recognize that this simplification does not fully capture the complexity of opioid use behaviors. Future studies could leverage novel repeated measures ML approaches to model transitions into opioid misuse to and from medically prescribed use on a daily or momentary basis (i.e., with ecological momentary assessment, see Bruneau et al., 2021; Frimerman et al., 2021) to provide deeper, more nuanced insights into the progression from appropriate use to misuse and vice versa.

For both the cognitive tasks, we observed that using information from the opioid cue alone was sufficient and results in better performance for detecting opioid misuse as compared to using pleasure and neutral cues. This finding is sensible in light of the incentive-sensitization theory of addiction, which proposes that drug-related cues become imbued with salience during the process of addiction (Berridge and Robinson, 2016). We also found that shorter analytic time windows (45 s) had the greatest predictive power, likely due to the possibility that shorter windows may be more sensitive to transient, event-related fluctuations in physiology and behavior, which may carry more discriminative information about opioid misuse than longer windows that wash out such effects. Additionally, we observed that behavioral signals, such as error rates, have greater predictive validity than physiological signals. Given that model predictions were based on responses to cognitive task data, this discovery opens the door for developing mobile, app-based digital biomarkers for opioid misuse risk detection in daily life. Moreover, future iterations of this technology could be used to predict treatment response and determine if a given patient requires a higher level of mental health services (e.g., intensive, evidence-based psychotherapy) to prevent the transition from opioid misuse to full-blown opioid use disorder.

### 4.1. Limitations

Importantly, the validity of our model was based on ground truth derived from a validated and widely used self-report measure of opioid misuse (COMM). Because some patients may be reluctant to self-report opioid misuse, reporting bias is possible. Thus, we may not have correctly classified all MISUSE+ participants in the sample. Nonetheless, we obtained a NIH certificate of confidentiality to encourage honest disclosure, and a majority endorsed opioid misuse on this measure. Nevertheless, it is important to emphasize that a primary goal of this study was to develop an objective measure of opioid misuse risk. Benchmarking our machine learning algorithm against a self-report measure does not enable us to determine the algorithm’s ability to detect actual misuse. Instead, it assesses how effectively the algorithm identifies a validated proxy of misuse (the COMM) with good accuracy that is already in clinical use. Future studies are needed to determine whether our model accurately predicts opioid misuse as triangulated by additional measures including drug urine screen and prescription drug monitoring program (PDMP) data.

Also, we trained and tested our model on a dataset with a higher proportion of MISUSE+ participants; however, in a real-world setting, opioid misuse is less common so the model needs retraining (or fine tuning) to learn real-world distributions. Participants were seated and stationary during cognitive tasks to ensure high-quality physiological signals. However, model performance is uncertain in scenarios where participants engage in activities like walking, limiting generalizability to mobile environments (Natarajan et al., 2016; Spathis et al., 2021).

Another important limitation is the sensitivity of the model, which was 0.68 in this study. This sensitivity level implies that some individuals who misuse opioids may not be identified, and conversely, there is a risk of misclassifying individuals as MISUSE+, potentially impacting their access to medical care. To address this concern, the model should not be used as a standalone diagnostic tool but as a component of a broader decision-making framework that includes clinical judgment and supplementary assessments. Future studies should focus on improving sensitivity through larger, more diverse datasets and feature refinement, while also exploring adaptive thresholds to balance sensitivity and specificity based on the clinical context.

Future studies could use our multivariate temporal fusion approach coupled with a robust multimethod assessment of opioid misuse to minimize false negatives and enhance the model’s applicability in real-world clinical settings.

### 4.2. Conclusion

By analyzing physiological signals and cognitive task data with advanced deep learning techniques, we developed an accurate opioid misuse detection system. Results from this study should be considered heuristic rather than confirmatory; additional research is needed to benchmark our machine learning system against objective measures of opioid misuse to ensure the replicability and robustness of its predictions. Nonetheless, these findings contribute to ongoing efforts in opioid management, emphasize potential benefits from leveraging digital healthcare and machine learning for developing predictive models of opioid misuse risk and other substance use disorders, and provide a foundation for future adaptive intervention approaches (Carpenter et al., 2020).

## Supplementary Material

1

## Figures and Tables

**Table 1:** Sample characteristics.

Characteristic		No. (%)

Age, Mean (SD)		50.6 (13.34)
Opioid Misuse Status	MISUSE-	53 (31.3)
	MISUSE+	116 (68.6)
COMM score^a^, Mean (SD)		15.0 (10.1)
Sex at Birth	Male	71 (42.0)
	Female	98(58.0)
Race/Ethnicity	White	118 (69.8)
	Black or African American	18(10)
	Hispanic	6 (3.5)
	American Indian or Alaska Native	6 (3.5)
	Asian	2 (1.1)
	Other	4 (2.3)
	Declined to answer	15 (8.8)
Yearly Income	< $20 K	58 (34.3)
	$20,001–40,000	48 (28.4)
	$40,001–60,000	15 (8.8)
	$60,001–80,000	10 (5.9)
	> $80,000	12 (7.1)
	Declined to answer	26(15.3)
Pain Conditions and Location^b^	Lower Back	105 (62.1)
	Arthritis	28 (16.5)
	Fibromyalgia	20 (11.8)
	Neuropathic	6 (3.5)
	Cervical Pain	17 (10)
	Migraine or tension headache	14 (8.2)
	Other	24 (14.2)
Opioid Prescription^b^	Oxycodone	56 (33.1)
	Hydrocodone	54 (31.9)
	Tramadol	32 (18.9)
	Morphine	16 (9.4)
	Buprenorphine	7 (4.1)
	Methadone	6 (3.5)
	Hydromorphone	3 (1.7)
	Other	13 (7.7)

a COMM=Current Opioid Misuse Measure; Score range, 0–68, with higher scores indicating greater likelihood of current opioid misuse.

b Some percentages sum to greater than 100 % because participants could report multiple pain conditions or locations and opioid prescriptions.

**Table 2:** Variable importance scores for static and continuous time variates.

(a) Static covariates				
Feature-type	Feature	Percentile Weights		
		10 %	50 %	90 %
**Cognitive**	**Task-type**	0.102	0.124	0.167
**Cognitive**	**Cue-type**	0.181	0.281	0.335
(b) Continuous covariates				
Feature-type	Feature	Percentile Weights		
		10%	50 %	90 %
**Sensory**	**ECG**	0.172	0.183	0.215
**Sensory**	**Respiratory**	0.062	0.068	0.083
**Cognitive**	**Reaction times**	0.165	0.196	0.224
**Cognitive**	**Error rates**	0.283	0.304	0.323

For (a) static covariates, we observed that cue type information contributed more predictive power than task type. For (b) continuous covariates, we observed that information from cognitive tasks to be more important than sensor data with error rates information contributing the most predictive power. To generate this table, we used all the available data instead of 10-fold validation. All the weights are normalized to facilitate interpretation.

## Data Availability

De-identified patient data in aggregate (suitable for meta-analysis) will be shared upon reasonable request with a signed and approved data sharing agreement submitted to Eric L Garland (egarland@health.ucsd.edu).

## Code availability

Code will be made freely available to qualified academic investigators for non-commercial research. Investigators may submit a request for code to Bhanu Teja Gullapalli (bgullapalli@ucsd.edu) or Tauhidur Rahman (trahman@ucsd.edu) who will grant permission to release the code.

## Appendix A. Supporting information

Supplementary data associated with this article can be found in the online version at doi:10.1016/j.drugalcdep.2025.112774.
