# Supplementary material for: Opioid misuse detection from cognitive and physiological data with temporal fusion deep learning
Source: Drug Alcohol Depend. Author manuscript; Available in PMC 2026 Jun 18. (PMC13277149; doi:10.1016/j.drugalcdep.2025.112774)
Supplement: 1 [file NIHMS2169502-supplement-1.docx]

## Machine Learning Model Development

Data collected from the study are divided using a 45-second time window, which is shifted by 20 seconds. The input to the model shown in Figure 2 is a 45-second time window that consists of sensor and cognitive data. We represent this data as Static: Cognitive Task type (Dotprobe/Go-NoGo) and Cue type (Opioid/Pain/..), which remain constant throughout this window; Continuous: ECG, Respiration, and Cognitive task performance (Reaction times and Error rates) that vary continuously based on user’s response and reaction in the study. Each second off this time window is used as a time step to the model. The input time steps are first passed through the variable selection network that extracts the most salient or informative features from the static and time dependent covariates for the prediction. The output from this network is combined with the embedding extracted from the static data using a static covariate encoder of Gated residual network (GRN), this combined data is passed through LSTM encoder units that extracts embeddings (or feature representations) for each time step.

Embeddings from all the time steps are combined through a self-attention mechanism to learn long-term relationships across different time steps. The self-attention mechanism operates by generating a matrix of attention scores through scaled dot-product operations between all pairs of time steps in the sequence. To enable diverse and robust representations, we utilized the multi-headed attention mechanism inherent to the TFT architecture. This mechanism, which is a core component of the TFT model, captures temporal relationships and feature interactions without requiring additional modifications to the original architecture. The output from the multi-headed attention is then passed through a GRN to incorporate additional temporal processing on the output embeddings. We integrated skip-connections at every aspect of the network to expedite convergence. Subsequently, the output from the GRN traverses a Fully-connected (FC) layer to compute the prediction probability of the user belonging to the MISUSE- class.

Given that our dataset contains more MISUSE- participants than MISUSE+ participants, we designated the former as the positive label. The model is trained from end-to-end using an objective loss function of weighted Cross-entropy between predicted and ground truth labels.For all the categorical variables (task type, cue type) we use entity embeddings to keep uniformity with the continuous ones. We implemented the TFT using an available open-source (<https://github.com/google-research/google-research/tree/master/tft>).

We chose the TFT for its ability to model complex temporal dependencies and integrate static and dynamic features. Although the experiment involved controlled and intentionally timed tasks, the temporal evolution of behavioral and physiological responses, such as reaction times and heart rate variability, remains critical. These temporal patterns reflect cognitive and attentional processes linked to opioid misuse and are highly informative for classification. The controlled timing enhances consistency in data collection, enabling TFT to capture meaningful variations. Furthermore, TFT's superior performance compared to other temporal models further supports its suitability for this study.

We implemented the TFT model using Python version 3.9 , Scikit-learn version 1.0.2 and Pytorch 2.0. Model training occurred on a Mac PC equipped with an Apple M1 Max chip that utilizes a 10-core CPU and a 16-core graphics processing unit, alongside 32GB of random access memory. The tuning of hyperparameters that includes window-size, sliding window, kernel size, dropout, etc,. (eTable2 in Supplement ) was done using a grid search randomly sampled 1,000 data points, to mitigate the computational expense. We had a large set of hyperparameters to tune including window size, sliding window, kernel size, dropout, number of heads, batch size, and gradient norm, making a full grid search with LOSO cross-validation computationally infeasible. To mitigate this issue, we randomly sampled 1,000 data points to identify faster-converging hyperparameter combinations, assuming these patterns would generalize to the full dataset. This strategy efficiently reduced the hyperparameter space while keeping computational costs manageable. We provide a description of the training process, and input features, and interpretability in Model Architecture in Figure 2.

## Statistical Results

We conducted a 2-way ANOVA to detect differences in wearable data and cognitive features due to cue type and opioid use class (MISUSE+ vs MISUSE-). For this analysis, we considered various time-, frequency-domain features of Heart rate (HR) and Respiration (Resp) and various statistical features of cognitive tasks. The complete list of these features is given in eTable 1A below.

| Modality | Features |
| --- | --- |
| Heart rate (HR) | HR NNI Counter, HR NNI Mean, HR NNI Min, HR NNI Max, HR Mean, HR Min, HR Max, HR Std, HR SDNN, HR RMSSD, HR NN50, HR PNN50, HR NN20 |
| Respiration (Resp) | Resp Mean, Resp RMSSD, Resp SDSD, Resp Phase Duration Inspiration,Resp Phase Duration Expiration, Resp LF, Resp HF, Resp LFHF |
| Cognitive | Reaction_time, Error_rates:- Go accuracy, NoGo accuracy, Dot Probe task accuracy |

eTable 1A: List of features considered for 2-way Anova. For all the cognitive features, we considered the mean statistical value from the whole task/cue-type.

Using the frequentist statistical approach, we found few statistically significant predictors of opioid misuse status. Specifically, among the time-, frequency-, and static (cognitive) features outlined in eTable 1A, we observed significant effects of the independent variables HR RMSSD, Resp Mean, and Resp RMSSD on the user groups categorized as MISUSE+ and MISUSE- classes (detailed results in eTable 1B). These effects were also only observed in the Dot Probe task. In contrast, the temporal deep learning approach yielded strong predictive power in identifying opioid misuse. Traditional frequentist statistics may fail to leverage the nonlinear and complex temporal patterns lying in the big data produced by cognitive tasks and psychophysiology. In contrast, our study used a temporal fusion-based multivariate machine learning approach, analyzing data across multiple timescales to improve the detection of opioid misuse (as described in the section titled “Results: Predictive Performance Comparison with specific Task and Cue type”).

| **Source** | **Dependent Variable** | **F** | **Sig.** |
| --- | --- | --- | --- |
| Cue Type | HR RMSSD | 0.197 | P = .899 |
| Opioid Use class | HR RMSSD | 5.123 | **P = .023** |
| Cue Type * Opioid Use class | HR RMSSD | 0.039 | P = .990 |
|  |  |  |  |
| Cue Type | Resp Mean | 0.109 | P = .955 |
| Opioid Use class | Resp Mean | 28.093 | **P < .001** |
| Cue Type * Opioid Use class | Resp Mean | 0.447 | P = .720 |
|  |  |  |  |
| Cue Type | Resp RMSSD | 0.356 | P = .784 |
| Opioid Use class | Resp RMSSD | 7.997 | **P < .01** |
| Cue Type* Opioid Use class | Resp RMSSD | 0.117 | P = .950 |

eTable 1B: The results of the 2-way Anova analysis. Statistical significant main effects are highlighted in bold font.

##

##

##

##

##

##

## Model Hyperparameters

For the reproducibility of our results, we are providing the model hyperparameters in the eTable 2.

| **Hyperparameter** | **Search Range** |
| --- | --- |
| Learning rate | 5e-4, 1e-4, 5e-3, 1e-3, 5e-2, 1e-2 |
| Dropout | 0.1, 0.2 ,0.4, 0.6 |
| Num. heads | 1, 4, 6 |
| Batch size | 8, 16, 32 |
| Max. gradient norm | 0.01, 1.0, 100.0 |

eTable 2: Hyperparameter search conducted for the model. The numbers highlighted in red represent the parameters ultimately chosen for faster convergence on a randomly selected dataset of 1,000 data points. To ensure robustness, the selection process was repeated with 1,000 datapoints ten times using different seeds

##

## Validating different machine learning models

We have trained and evaluated several different state of the art machine learning models that will predict opioid misuse from the multimodal sensor time series data. The Temporal Fusion Transformer (TFT) model outperforms the other state of the art deep learning models and achieves an AUC-ROC of 0.77.

| **Model** | **AUC-ROC** | **Specificity** | **Sensitivity** |
| --- | --- | --- | --- |
| **TFT** | **0.77 (95% CI, 0.71-0.83)** | **0.84 (95% CI, 0.76-0.92)** | **0.68 (95% CI, 0.62-0.73)** |
| Bi-LSTM | 0.57 (95% CI, 0.47-0.69) | 0.55(95% CI, 0.57-0.66) | 0.59 (95% CI, 0.57-0.60) |
| GRU | 0.64 (95% CI, 0.57-0.69) | 0.70 (95% CI, 0.62-0.77) | 0.62 (95% CI, 0.58-0.66) |
| TCN | 0.60 (95% CI, 0.57-0.62) | 0.64 (95% CI, 0.60-0.66) | 0.67 (95% CI, 0.64-0.71) |

eTable 3: Predicting MISUSE- using Various Time-Series Models. The results utilize all data points from both tasks.
